# Supplementary material for: Temporal Genetic Dynamics of an Experimental, Biparental Field Population of Phytophthora capsici
Source: Front Genet. 2017 Mar 13;8:26. doi: 10.3389/fgene.2017.00026 (PMC5347166; doi:10.3389/fgene.2017.00026)
Supplement: Supplementary file 3 [file Data_Sheet_3.pdf]

Supplementary Text for

Temporal Genetic Dynamics of an Experimental, Biparental Field Population of *Phytophthora capsici*

Authors: Maryn O. Carlson, Elodie Gazave, Michael A. Gore, Christine D. Smart

Correspondence to: Michael A. Gore (mag87@cornell.edu) and Christine D. Smart (cds14@cornell.edu)

Table of Contents

Correcting for mitotic loss of heterozygosity (LOH) in Mendelian error (ME) estimates 2

Evidence for LOH events 2

Mating type associated SNPs in the field inbred 6

References 7

Correcting for mitotic loss of heterozygosity (LOH) in Mendelian error (ME) estimates
Initially, the mean proportion MEs for the *in vitro* F_1 (5.77%) and empirically defined field F_1 isolates (6.34%) exceeded our estimated genotyping error rate of 3% (by 2.77% and 3.34%, respectively). Motivated by this observation, we assessed whether specific SNPs were contributing disproportionately to overall ME estimates, by calculating the proportion MEs for each SNP in the combined *in vitro* F_1 and field F_1 subpopulations (n=143). An isolate with a proportion MEs exceeding the *in vitro* F_1 mean by three standard deviations (>10.65%) was classified as field inbred and otherwise as field F_1. The maximum number of tested sites for an individual consisted of the consensus parental genotypes excluding double heterozygous SNPs data, which are uninformative in assessing MEs, and SNPs with missing parental data.

As a result, we identified 848 ME-enriched SNPs, defined as a SNP with greater than 10% MEs (equivalent to 15 isolates with a ME). While some ME-enriched SNPs were isolated and randomly distributed throughout the genome, ME-enriched SNPs appeared in clusters in several instances (Supplementary Figure S6), suggestive of underlying biological factors rather than sequencing or genotyping error. We show below that these events occurred post-field inoculation but prior to genotyping of the parents, resulting in homozygous parental genotypes discordant with segregation of four haplotypes in the field population. Additionally, ME-enriched regions were associated with differentiation between the *in vitro* F_1 and field progeny in six of the seven cases.

Evidence for LOH events
The *in vitro* F_1 were representative of the field F_1 in terms of inbreeding coefficient distribution, site heterozygosity (data not shown) and MAF distributions (Supplementary Figure S5). To further evaluate the extent to which the *in vitro* F_1 equated to the field F_1, we performed a Fisher's exact test of allele frequency differences [1] between these two subpopulations at each SNP. We utilized a Fisher's exact rather than chi-square test due to the 0.08% of cases where the expected allele counts were <5 in all pairwise comparisons.

Genome-wide, allele frequencies between the *in vitro* F_1 and the field F_1 were similar, except for six regions (in scaffolds 8, 19, 26, 33, 35, 55) with 5% to 68% of SNPs within each region exceeding the multiple-test correction threshold (Supplementary Table S3 and Figure S7). These six regions were likewise highly differentiated between the *in vitro* F_1 and field inbred subpopulations (Supplementary Figure S7). Regions of differentiation co-localized with the identified ME-enriched SNP clusters (Supplementary Figure S8).

For each region, we first performed PCA on all isolates and the consensus parental genotypes, using only the SNPs within the minimum and maximum significantly differentiated SNPs in each scaffold. In each PCA, we observed four primary clusters, indicative of four distinct genotypes (Supplementary Figure S9). This was in accordance with expectations for an F_1 derived from heterozygous parents, where four segregating haplotypes result in four genotypes in the progeny. However, we observed very low heterozygosity in either or both the parents in these regions, suggesting fewer than four founding haplotypes (Supplementary Figure S8).

To understand the discrepancy between parental homozygosity and observed segregation in the field F_1, we locally phased all isolates in each region, excluding R-19, utilizing a deterministic

approach (see Methods). We then counted the number of distinct haplotypes in both the parents and the field F_1. The field F_1 presented four haplotypes (H1, H2, H3 and H4) in each region, whereas the parental genotypes provided evidence for only two to three haplotypes (Supplementary Table S4). For example, in the scaffold 26 region (R-26), if the parental isolates used to inoculate the field were as sequenced (A1 parent=H1/H2 and A2 parent=H3/H3), we would expect only H1/H3 and H2/H3 progeny. Yet, we observed H1/H4 and H2/H4 genotypes in the field F_1 and the *in vitro* F_1, resulting in an excess of MEs in R-26 for both the field F_1 and *in vitro* F_1 (Supplementary Figures S8 and S10). The simplest explanation for this discrepancy was that the sequenced A2 parental isolate underwent a mitotic LOH event in R-26 after the field inoculation and collection of *in vitro* progeny (i.e. during culture prior to sequencing). Thus, in R-26, the genotype of the sequenced isolate (H3/H3) differed from the inferred genotype (H3/H4) of the isolate used to found the field and *in vitro* subpopulations. Segregation in the scaffold 8 region (R-8) followed a similar pattern to segregation in R-26 (Supplementary Table S4 and Figure S8), consistent with both scaffolds being adjacent in linkage group 8 [2].

In the scaffold 19, 33, 35, and 55 regions, the *in vitro* F_1, with one exception, were present in only two of the four major PCA clusters (Supplementary Figure S9), and lacked one haplotype (H2) relative to the field F_1 (Supplementary Table S4 and Figure S12). Akin to R-8 and R-26, A1 parent homozygosity (H1/H1) in these four regions conflicted with the four observed genotypes, supporting incidence of LOH in the A1 parental culture in these regions. As the most parsimonious explanation for the presence of H2 in the field F_1, but not the *in vitro* F_1, we hypothesized that the A1 parental LOH event occurred in culture after the field inoculation (2008), but predated collection of the *in vitro* progeny (2010). This hypothesis was supported by markedly higher proportions of MEs in the field F_1 relative to the *in vitro* F_1 in the scaffold 19, 33, 35, and 55 regions (Supplementary Figure S8). Observation of the same pattern in these four regions, was consistent with presence of the corresponding scaffolds in linkage group 16 of the published linkage map [2].

In R-35 both the A1 and A2 parents were homozygous (H1/H1 and H3/H3, respectively), which would result in only H1/H3 progeny. Here, since we observed haplotype H4 (A2 parent haplotype) in both the *in vitro* and the field F_1, the A2 parent LOH event would have necessarily occurred post field inoculation and *in vitro* collection. To investigate the timing of this LOH event, we used the fact that the parental replicates represented multiple distinct culture time points (from 2013 to 2014), to compare the genotypes of the parental isolates in R-35 across culture time. The earliest cultures of the A2 parent (Supplementary Table S2) were heterozygous in R-35, recapitulating the inferred H3/H4 founding genotype (Supplementary Figure S13). Sequence-based evidence of the "ancestral" genotype strongly supported incidence of an LOH event during culture passage in 2014, after establishment of the field population and *in vitro* collection. All A1 replicates were homozygous in R-35, consistent with the A1 parent LOH event predating *in vitro* collection (2010). We also found evidence for heterozygosity in the earliest sequenced cultures of the A2 parental isolate in R-55 and in scaffold 7, associated with incidence of MEs in the *in vitro* F_1 and field F_1 (data not shown). Sequence based evidence and/or discordant segregation among *in vitro* and field progeny with respect to the parental genotypes associated with ME clusters, support mitotic LOH in the parental cultures as the explanation for incidence of heightened MEs in all seven ME-enriched SNP clusters.

Apart from the incidence of parental LOH in these regions, we observed skewed segregation ratios in both the field F_1 and *in vitro* F_1 in each of the six regions (Supplementary Table S4). In fact, distorted segregation drove allele frequency differences between these two subpopulations in R-8 and R-26, rather than the incidence of parental LOH events (as was the case in the other four regions). Further, four to eight *in vitro* F_1 isolates (Supplementary Figures S10 and S12) were homozygous in each region (Supplementary Table S4). (Homozygous isolates were removed from calculation of MEs presented in Supplementary Figure S8.) These specific instances of anomalous segregation, i.e. haplotype homozygosity in an F_1, could result from mitotic LOH, as likely occurred in the parental isolates due to serial culturing, or by non-Mendelian meiotic processes. In rare cases (3 to 5 isolates), we also observed homozygosity in the field F_1 in these regions (Supplementary Table S4 and Figures S10-S12). As the number of cultures prior to sequencing was not controlled, we could not infer the relative frequency of meiotic or mitotic LOH events in the field F_1 versus *in vitro* F_1 isolates. In addition, the smaller *in vitro* F_1 sample size (n=41) may have influenced differentiation from the field F_1 in R-8 and R-26.

Due to the low frequency (<5%) of haplotype homozygosity among field F_1 isolates in these regions, aberrant LOH processes likely minimally influenced our analysis. The instances of LOH in the A1 parent that manifested in allele frequency differences between the *in vitro* F_1 and field F_1 (Rs-19, 33, 35, and 55), reflected the genomic changes occurring in culture from the time of field inoculation to collection of the *in vitro* progeny, 2008 to 2010. These four LOH tracts spanned less than 1 Mb (approximately 2% of the ~48 Mb analyzed), supporting negligible influence of large scale LOH events on our genome-wide analyses.

Mating type associated SNPs in the field inbred

Based on the 184 SNPs associated with mating type in the F_1, the PCA of all isolates (*in vitro* and field; n=203) showed incomplete differentiation according to mating type (Supplementary Figure S16). When the Fisher's exact test was repeated in the field inbred subpopulation (n_{A1}=21 and n_{A2}=32), only SNPs within scaffolds 4 and 27 (n=53 and 20, respectively) were significantly differentiated (Supplementary Table S8 and Figure S15). Utilizing only the intersection of significant SNPs, from both field F_1 and field inbred tests (n=51), the PCA more discretely separated isolates by mating type, revealing two primary clusters (Supplementary Figure S16). This result suggested that only a subset of the F_1 differentiated SNPs were tightly linked to the mating type determining factor, but also may have been influenced by reduced power due to the smaller field inbred sample size.

References

1. Weir BS. Genetic Data Analysis II. Sunderland, MA: Sinauer Associates, Inc. Publishers; 1996.
2. Lamour KH, Mudge J, Gobena D, Hurtado-Gonzales OP, Schmutz J, Kuo A, et al. Genome Sequencing and Mapping Reveal Loss of Heterozygosity as a Mechanism for Rapid Adaptation in the Vegetable Pathogen *Phytophthora capsici*. MPMI. 2012;25: 1350–1360. doi:10.1094/MPMI-02-12-0028-R
