# Supplementary material for: Temporal Genetic Dynamics of an Experimental, Biparental Field Population of Phytophthora capsici
Source: Front Genet. 2017 Mar 13;8:26. doi: 10.3389/fgene.2017.00026 (PMC5347166; doi:10.3389/fgene.2017.00026)
Supplement: Supplementary file 4 [file Data_Sheet_4.pdf]

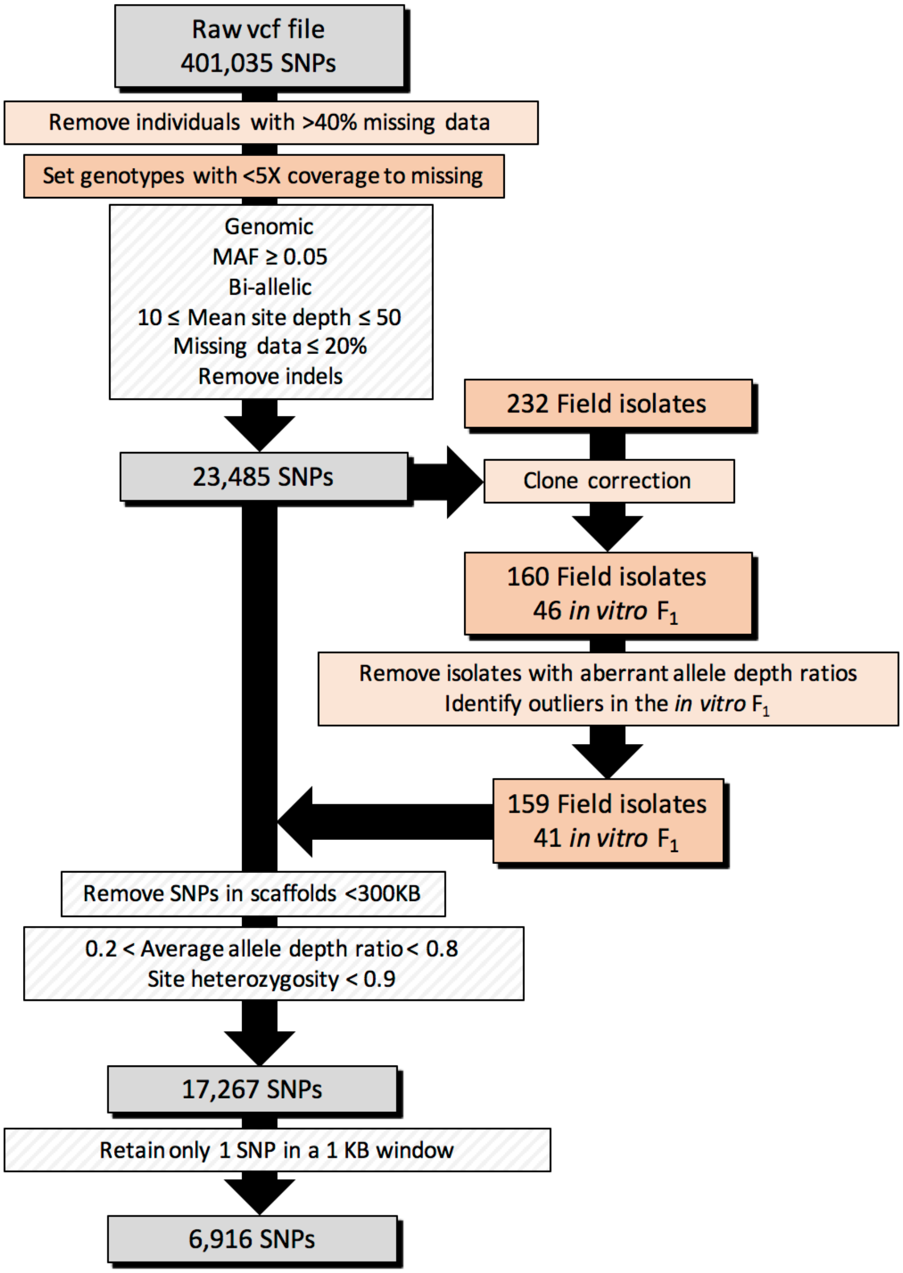

Figure S1. SNP and individual filtering pipeline. Gray and white shaded boxes indicate SNP filtering steps. Orange shaded boxes indicate individual filtering steps.

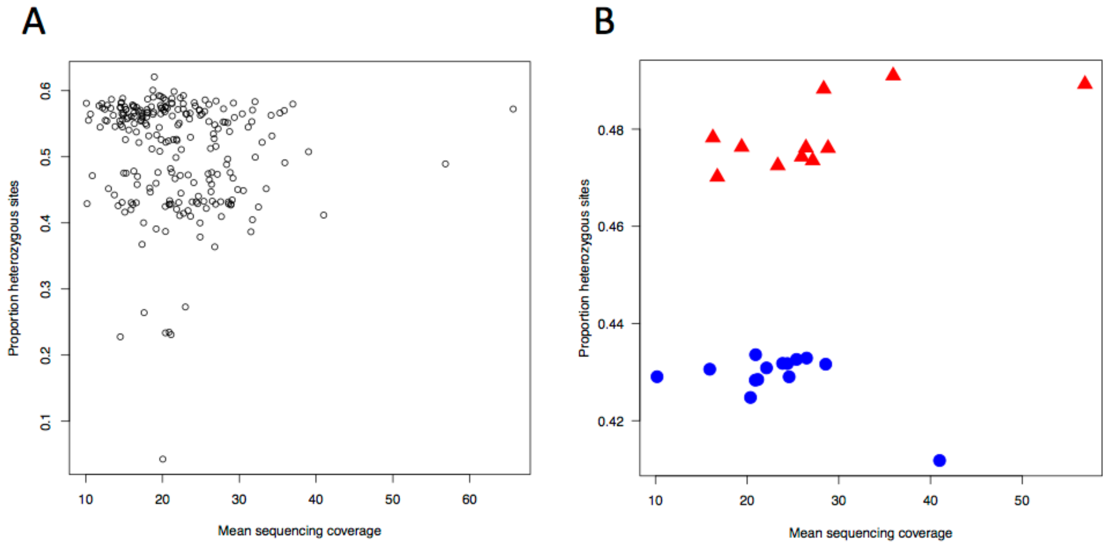

Figure S2. Relationship between sequencing coverage and heterozygosity. The proportion of heterozygous genotypes per sample plotted against individual mean sequencing coverage (*n*=23,485 SNPs). A) For all samples prior to clone-correction and outlier removal; and B) for only replicates of the A1 parental isolate (*n*=14; blue circles) and the A2 parental isolate (*n*=11; red triangles).

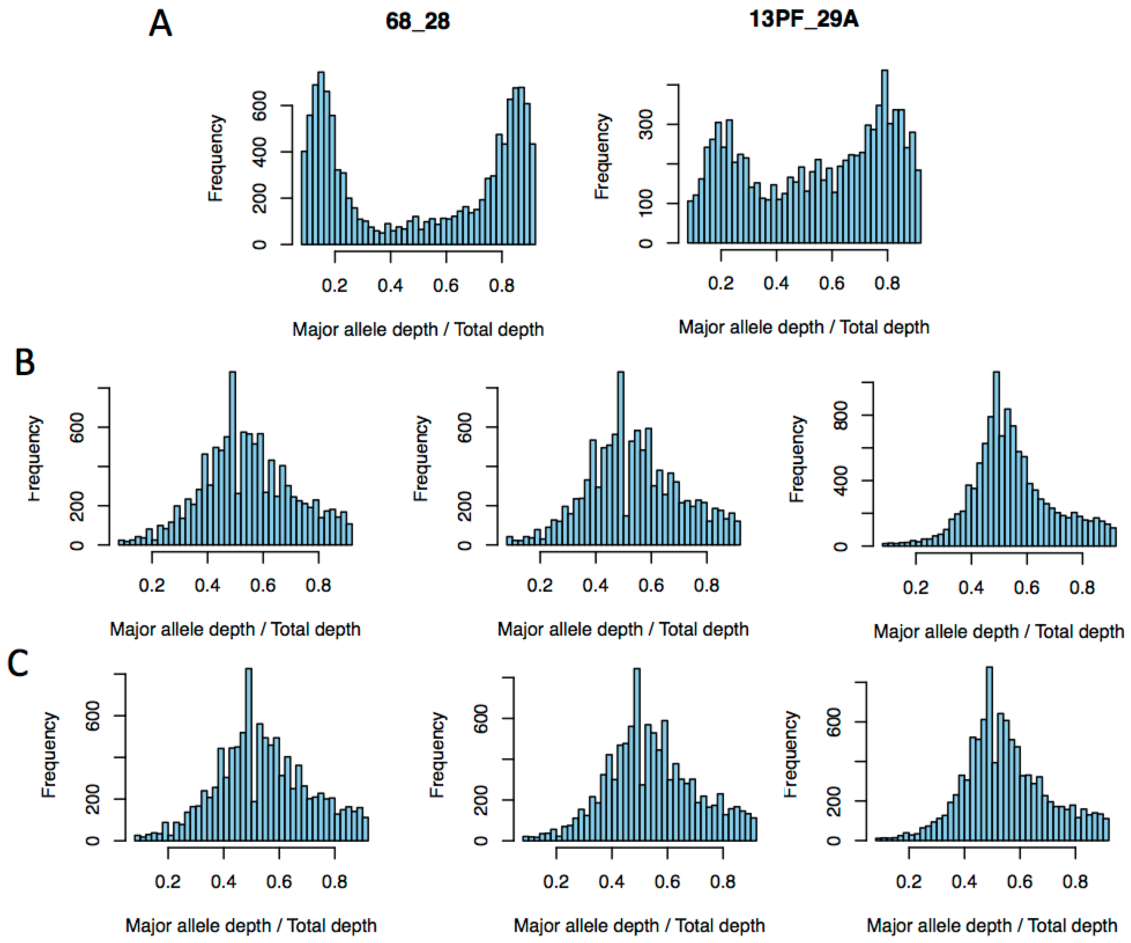

Figure S3. Skewed allele depth ratios in two isolates suggest ploidy variation. Histograms of the ratio of the major allele depth to the total depth for each heterozygous genotype for each isolate (at 23,485 SNPs). A) One *in vitro* and one field isolate display grossly aberrant allele depth ratios suggestive of ploidy variation. In contrast, allele depth ratios for the (B) A1 and (C) A2 parental isolates (three replicates each) were centered at approximately 0.5.

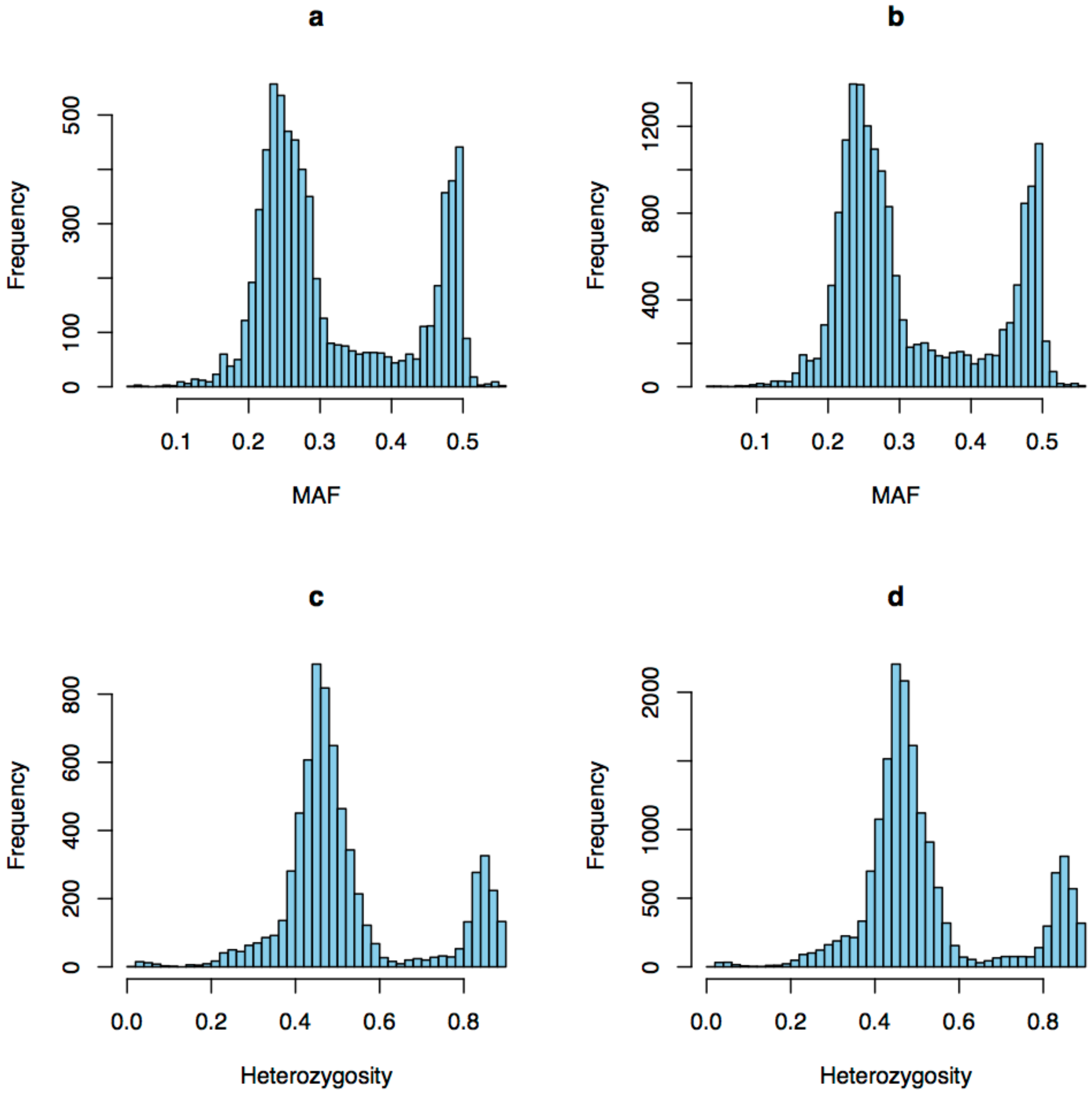

Figure S4. Comparing pruned and unpruned data sets. Minor allele frequency (MAF) and heterozygosity distributions for the unpruned (*n*=17,267) and pruned SNPs (*n*=6,916) in the field population (*n*=159 isolates). (A) and (C) are for the pruned data set. (B) and (D) are for the unpruned data set.

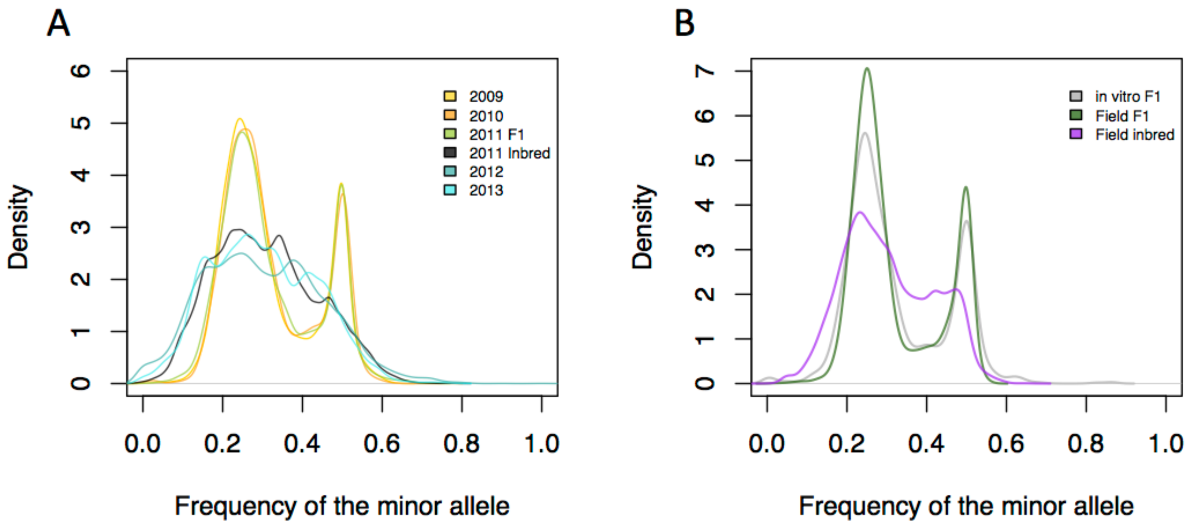

Figure S5. Minor allele frequency (MAF) distributions for the field and *in vitro* populations. A) MAF distributions for each year in the field population. Within years containing F_1 isolates, we observe peaks at 0.25 and 0.5, consistent with expectations for a population derived from only two parents. The MAF distribution of F_1 isolates in 2011 was similar to the MAF distributions in 2009 and 2010, years which contained exclusively F_1 isolates. B) The field F_1 subpopulation MAF distribution was consistent with the that of the *in vitro* F_1. The field inbred MAF distribution deviated from expectations for an F_1, denoting allele frequency changes.

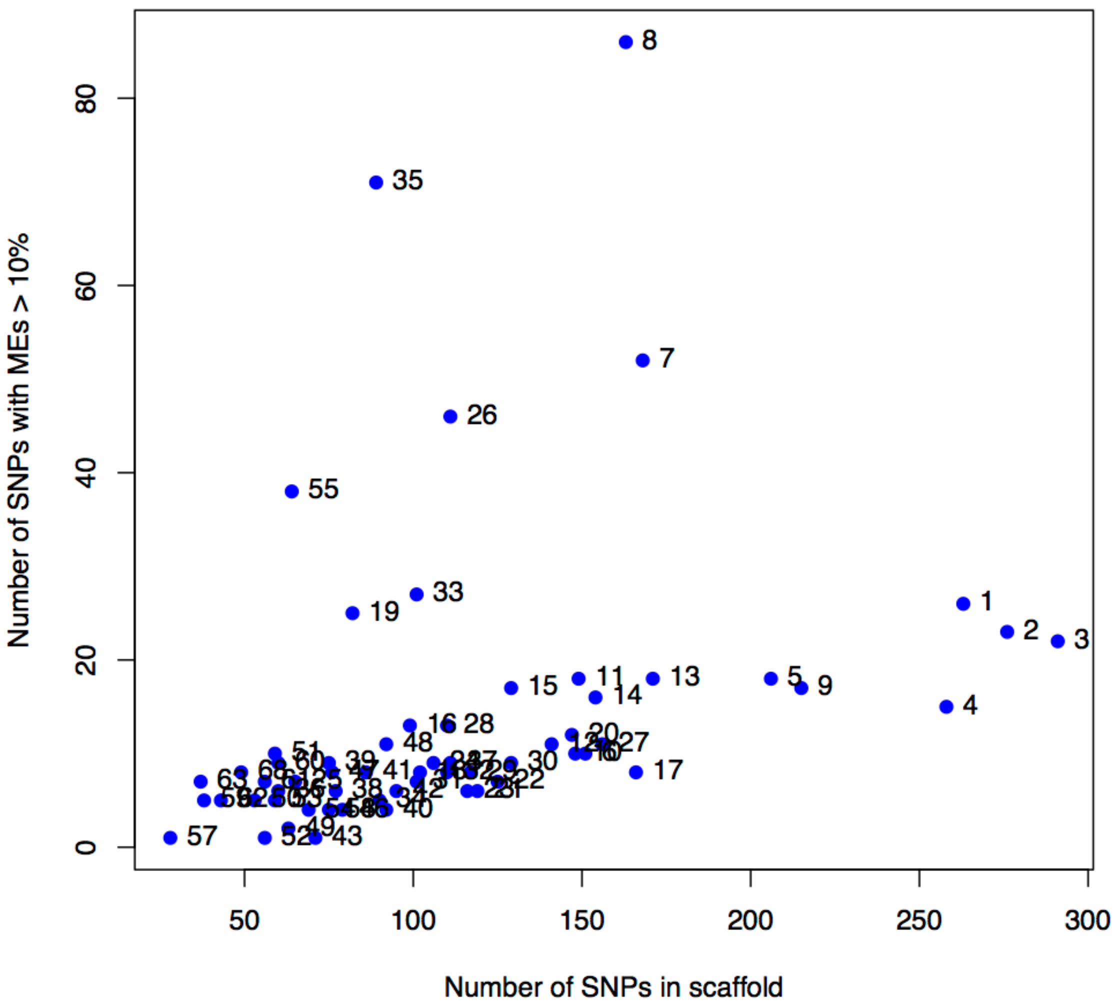

Figure S6. Relationship between number of SNPs in each scaffold and the incidence of Mendelian error (ME) enriched SNPs among the *in vitro* F_1 and empirically defined field F_1 (*n*=143) prior to removal of ME enriched SNPs. SNPs enriched for MEs were defined as SNPs where greater than 10% of *in vitro* F_1 and field F_1 isolates had a ME (at least 15 isolates). The number of ME enriched SNPs was plotted as a function of the number of SNPs in each scaffold, identifying seven scaffolds (7, 8, 19, 26, 33, 35, and 55) with excess ME-enriched SNPs relative to the other scaffolds. Data points are labeled with the scaffold number.

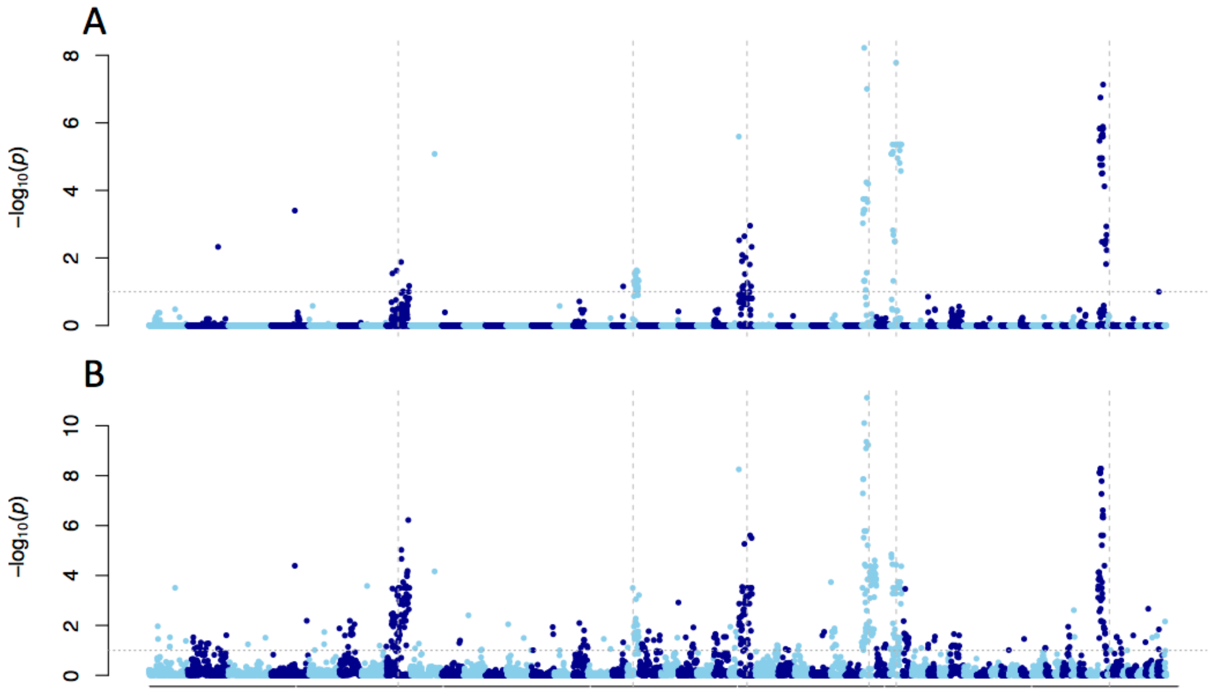

Figure S7. Regions of differentiation between the *in vitro* F_1 and the field F_1 and field inbred subpopulations identified using Fisher's exact tests of allele frequency differences at each SNP. Negative $\log_{10}$-transformed, false-discovery rate (FDR) adjusted *P*-values from pairwise comparisons between the (A) *in vitro* F_1 and field $F_{1;}$ and (B) *in vitro* F_1 and field inbred plotted for each SNP. SNPs are ordered relative to physical position and colors alternate by scaffold. Gray vertical dashed lines in A-C indicate scaffolds pertaining to regions of differentiation between the *in vitro* F_1 and the field F_1. The gray dotted line in A and B denotes the 10% FDR threshold.

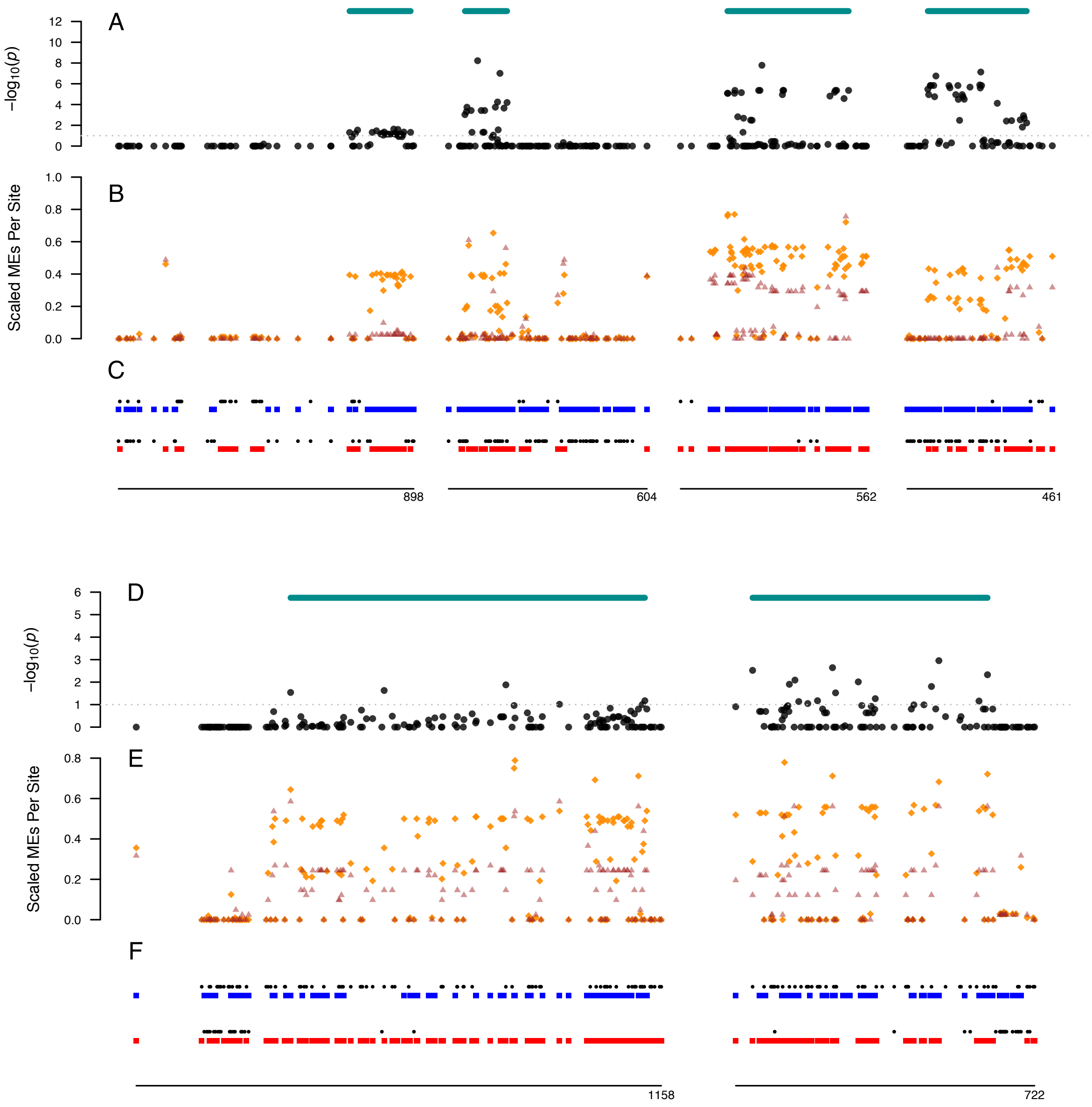

Figure S8. Regions of differentiation between the *in vitro* F_1 and field F_1 were associated with loss of heterozygosity (LOH) events in the parental cultures. A) and D) show the negative log_{10}-transformed, FDR adjusted *P*-values from the Fisher's exact test of allele frequency differences between the *in vitro* F_1 and field F_1, relative to physical position (kb), in scaffolds corresponding to regions of differentiation. The teal bars span each differentiated region. B) and E) show the proportion of individuals with a Mendelian error (ME) for each SNP in the *in vitro* F_1 (brown triangles) and the field F_1 (orange diamonds), excluding homozygous isolates. C) and F) are the parental genotypes represented by blue (A1 parent) and red (A2 parent) squares for homozygous genotypes and black dots for heterozygous genotypes. A-C show, in order, scaffolds 19, 33, 35, and 55. And, D-F show, in order, scaffolds 8 and 26.

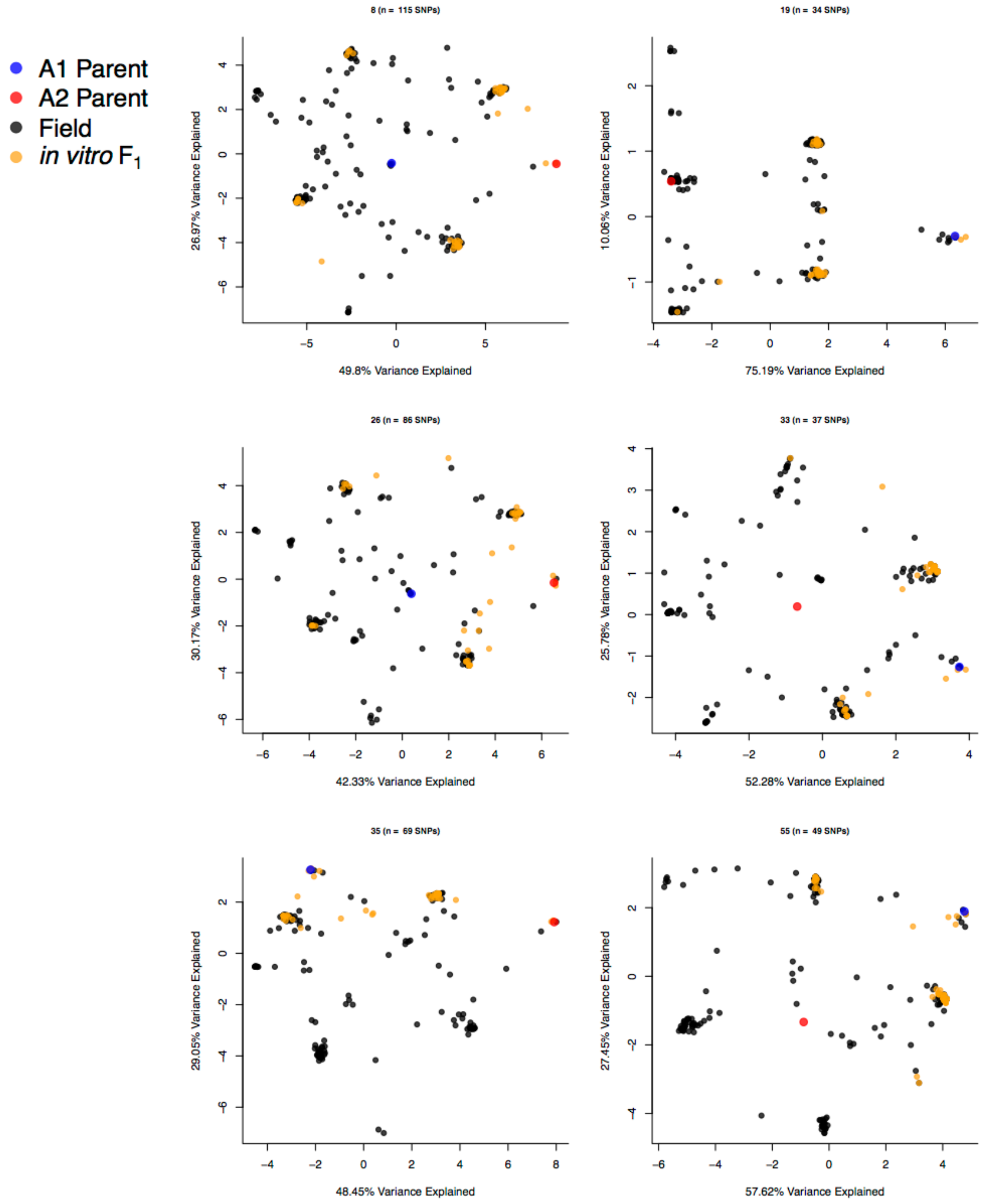

Figure S9. Principal component analysis (PCA) in scaffolds pertaining to regions of interest (ROIs). PCA was performed on the *in vitro* F_1, field F_1, and the field inbred isolates, as well as, the consensus parental genotypes with only SNPs in each of the six differentiated regions. All PCAs show four primary clusters, corresponding to four genotypic classes. The field isolates (*n*=159) are represented by filled, black circles. The *in vitro* F_1 (*n*=41) are represented by orange, filled circles. The A1 and A2 consensus parental genotypes are represented by blue and red filled circles, respectively.

A

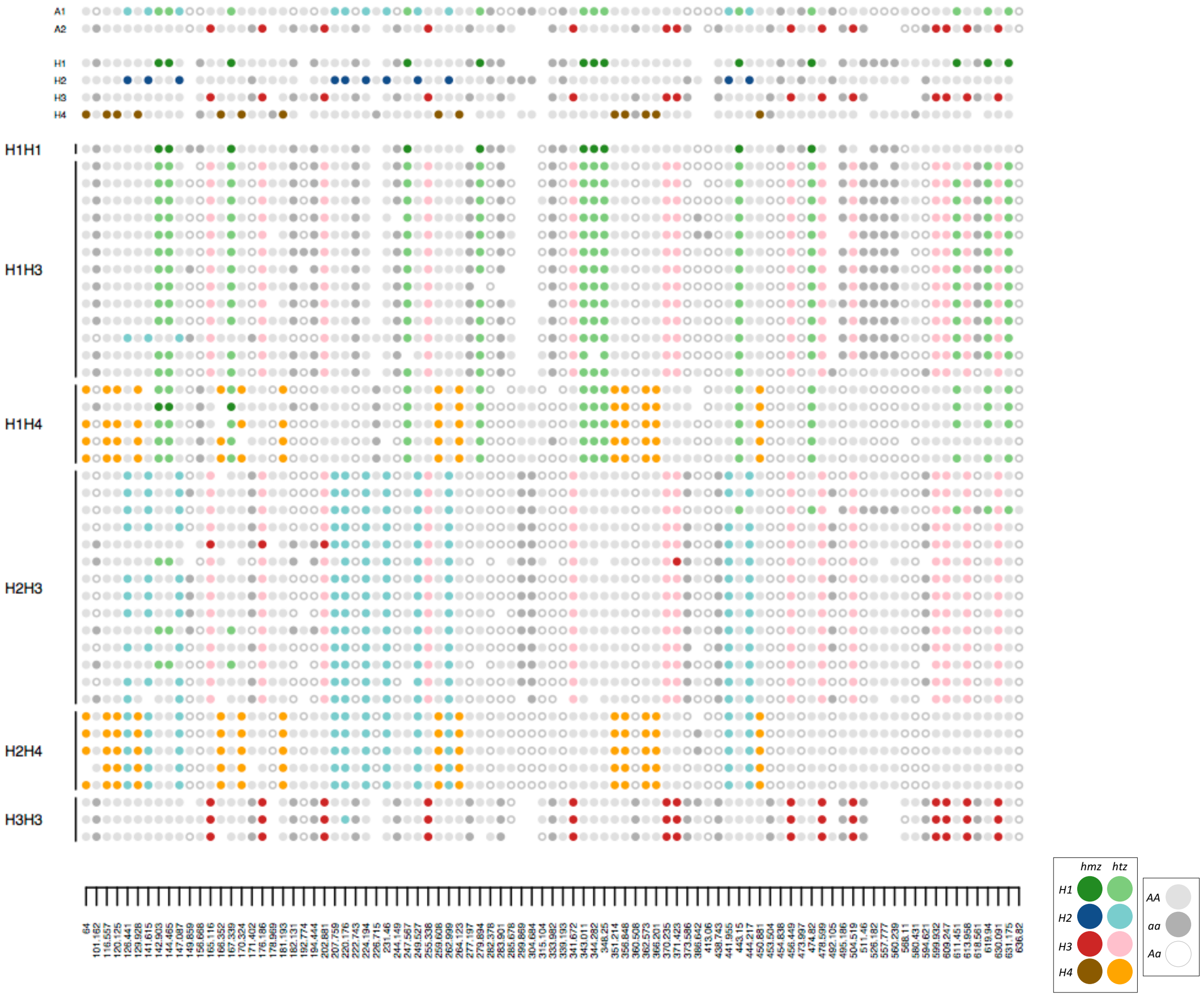
A1
A2
H1
H2
H3
H4
H1H1
H1H3
H1H4
H2H3
H2H4
H3H3
hmz
htz
H1
H2
H3
H4
AA
aa
Aa

B

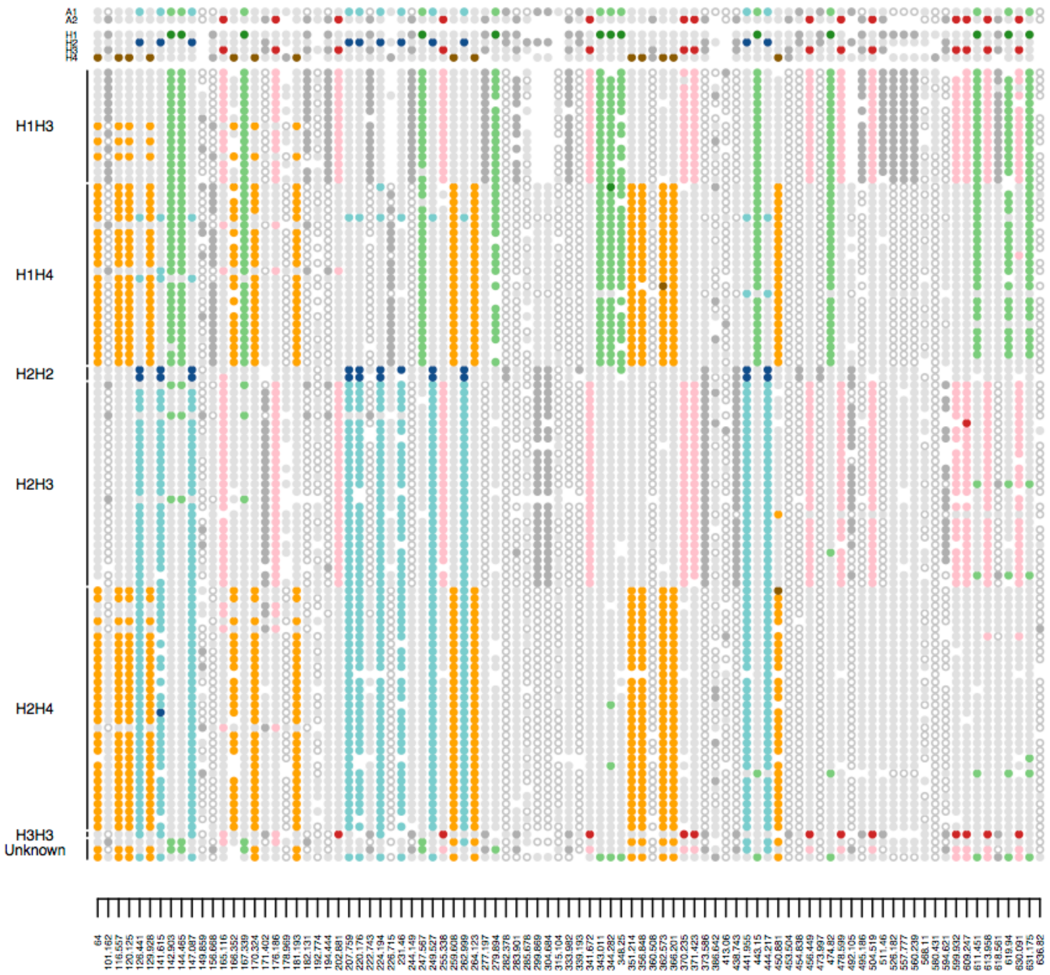

Figure S10. Phase diagram for R-26 (scaffold 26). Haplotype tagging SNPs were identified from the phased parental genotypes, and each isolate genotype was represented with respect to these tagging SNPs (see Methods). Filled, colored circles indicate haplotype tagging SNPs, with darker colors indicating the homozygote state, and lighter shades indicating the heterozygous state. Filled, gray circles indicate homozygous genotypes at non-tagging SNPs, whereas open, gray circles indicate heterozygous genotypes at non-tagging SNPs (see legend). A1 parental haplotypes are indicated by H1 and H2. A2 parental haplotypes are indicated by H3 and H4. Missing genotypes are denoted by the absence of a circle. Phase diagrams for the parental isolates (top), identified haplotypes (middle) and (A) *in vitro* F_1 and (B) field F_1.

A

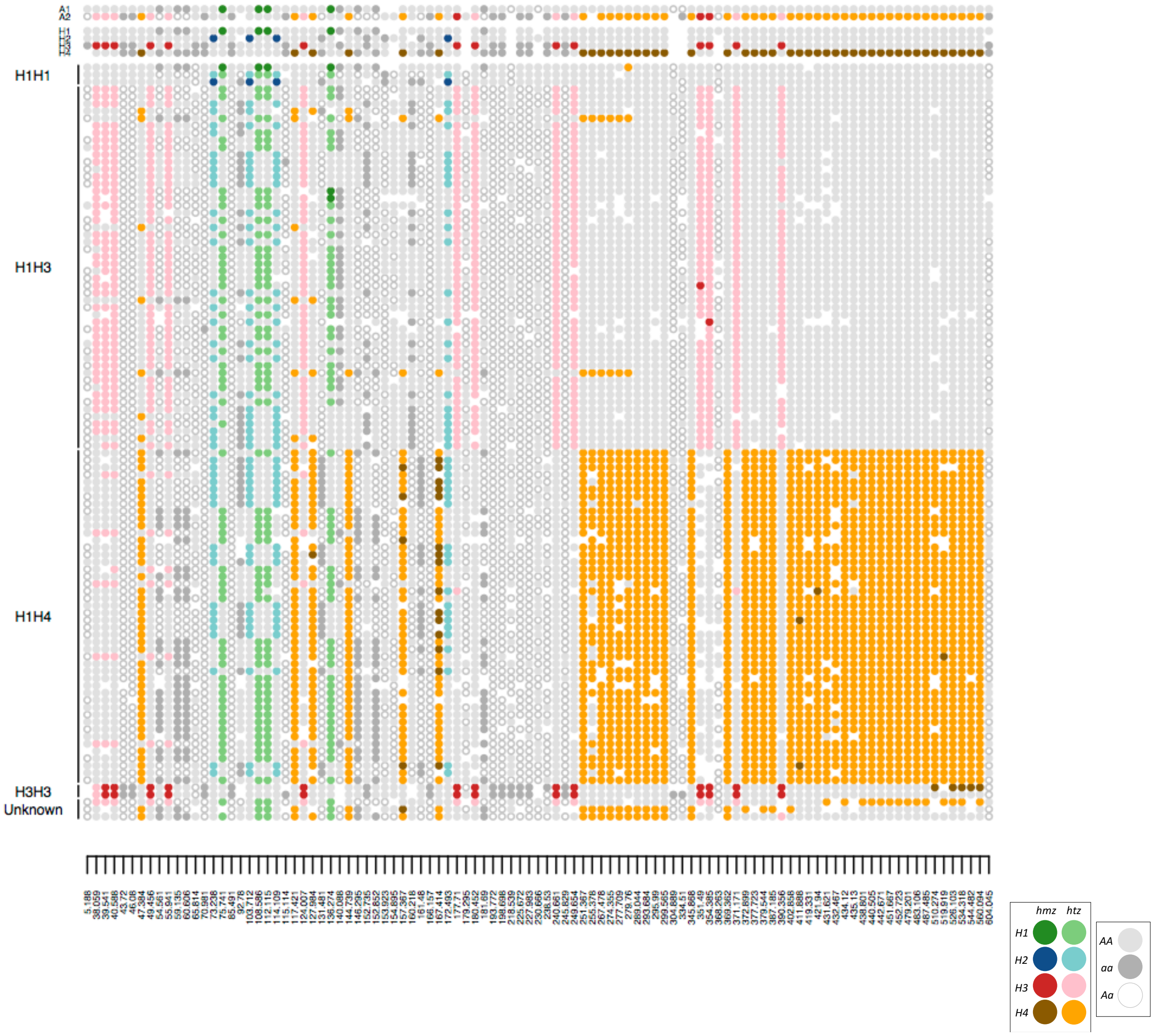

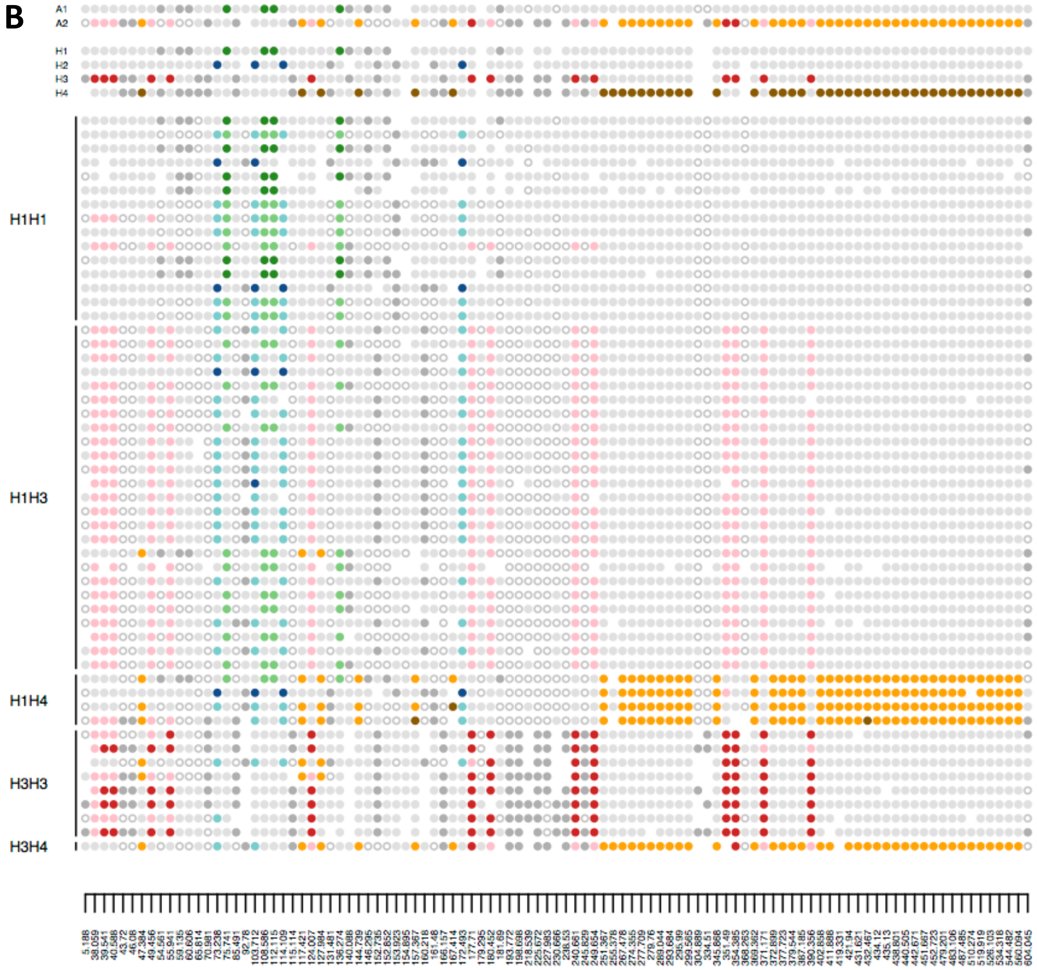

Figure S11. Phase diagram for ROI-1 (scaffold 33). Labeled genotypes based on ROI-1 only. Haplotype tagging SNPs were identified from the phased parental genotypes, and each isolate genotype was characterized with respect to the tagging SNPs (see Methods). Filled, colored circles indicate haplotype tagging SNPs, with darker colors indicating the homozygote state, and lighter shades indicating the heterozygous state. Filled, gray circles indicate homozygous, non-tagging genotypes, whereas open, gray circles indicate heterozygous non-tagging genotypes (see legend). Missing genotypes are denoted by the absence of a circle. A1 parental haplotypes are indicated by H1 and H2. A2 parental haplotypes are indicated by H3 and H4. Phase diagrams for the parental isolates (top), identified haplotypes (middle) and (A) field F_1 and (B) field inbred (bottom).

A

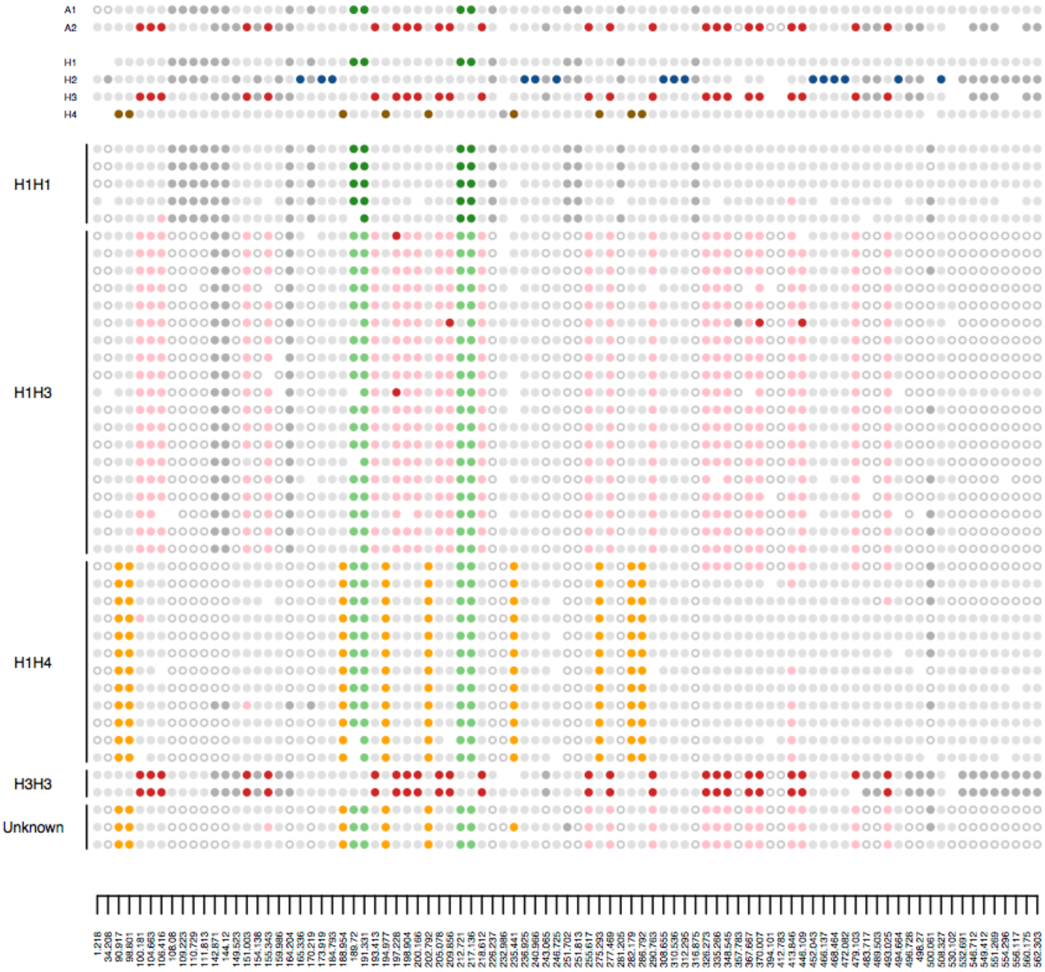
A1
A2
H1
H2
H3
H4
H1H1
H1H3
H1H4
H3H3
Unknown
1,218
34,208
90,917
98,801
100,181
104,663
106,416
108,08
109,223
110,729
111,813
142,871
144,12
149,523
151,003
154,138
155,343
159,986
164,204
165,336
170,219
173,919
184,793
188,954
189,72
191,331
193,413
194,977
197,228
198,904
200,166
202,792
205,078
209,856
212,721
217,136
218,612
226,237
232,986
235,441
238,925
240,966
243,065
246,725
251,702
251,813
255,617
275,293
277,469
281,205
282,179
286,792
290,763
308,655
310,536
312,295
316,875
326,273
335,286
348,545
357,783
367,667
370,007
394,101
412,783
413,846
446,109
452,043
466,137
468,464
472,082
479,103
483,717
489,503
493,025
494,664
496,728
498,27
500,061
508,327
530,102
532,691
546,712
549,412
551,289
554,296
556,117
560,175
562,303

B

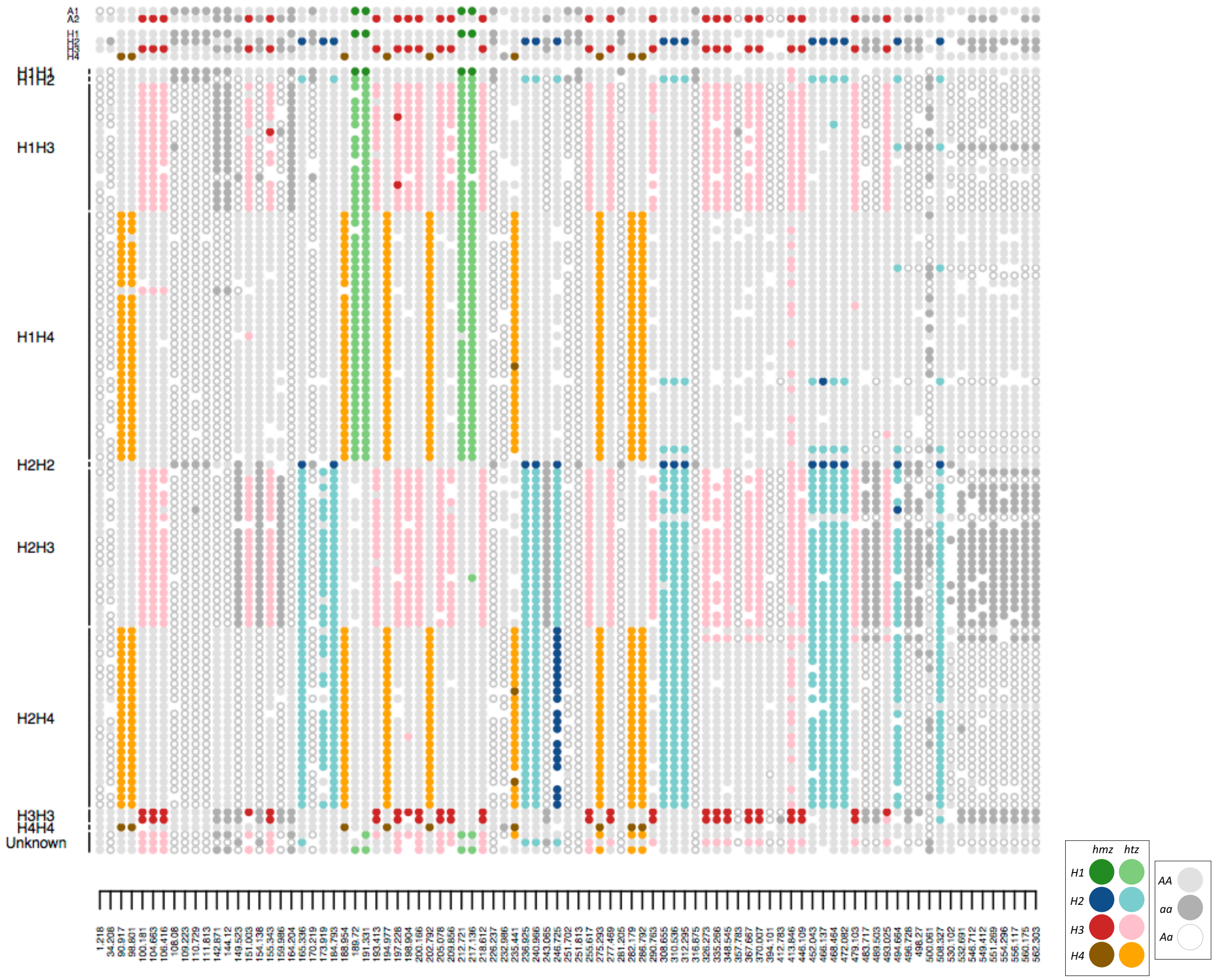

Figure S12. Phase diagram for R-35 (scaffold 35). Haplotype tagging SNPs were identified from the phased parental genotypes, and each isolate genotype was characterized with respect to the tagging SNPs (see Methods). Filled, colored circles indicate haplotype tagging SNPs, with darker colors indicating the homozygote state, and lighter shades indicating the heterozygous state. Filled, gray circles indicate homozygous, non-tagging genotypes, whereas open, gray circles indicate heterozygous non-tagging genotypes (see legend). A1 parental haplotypes are indicated by H1 and H2. A2 parental haplotypes are indicated by H3 and H4. Missing genotypes are denoted by the absence of a circle. Phase diagrams for the parental isolates (top), identified haplotypes (middle) and (A) *in vitro* F_1 (B) field F_1.

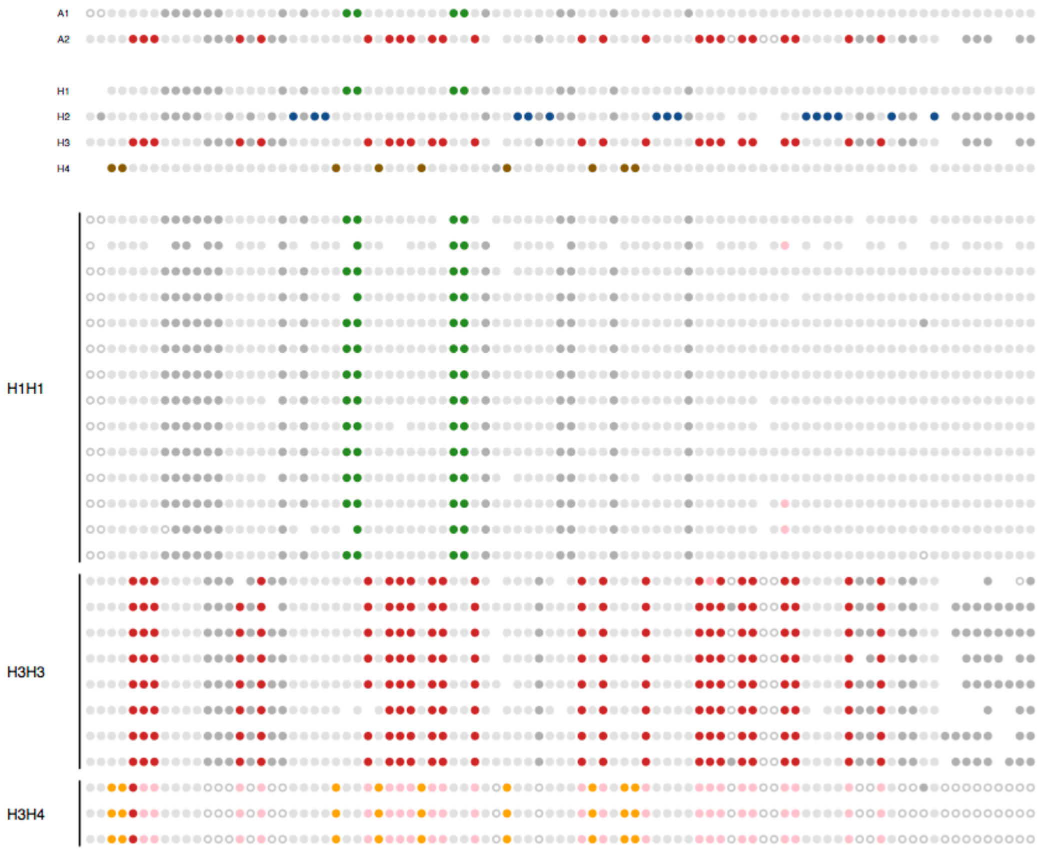

Figure S13. Phase diagrams for the parental replicates in R-35 (scaffold 35). All A1 parental replicates were H1/H1. The three A2 parental replicates sequenced prior to 2014 were H3/H4, whereas the later sequenced replicates were H3/H3 (see Supplementary Table S2). A1 parental haplotypes are indicated by H1 and H2. A2 parental haplotypes are indicated by H3 and H4.

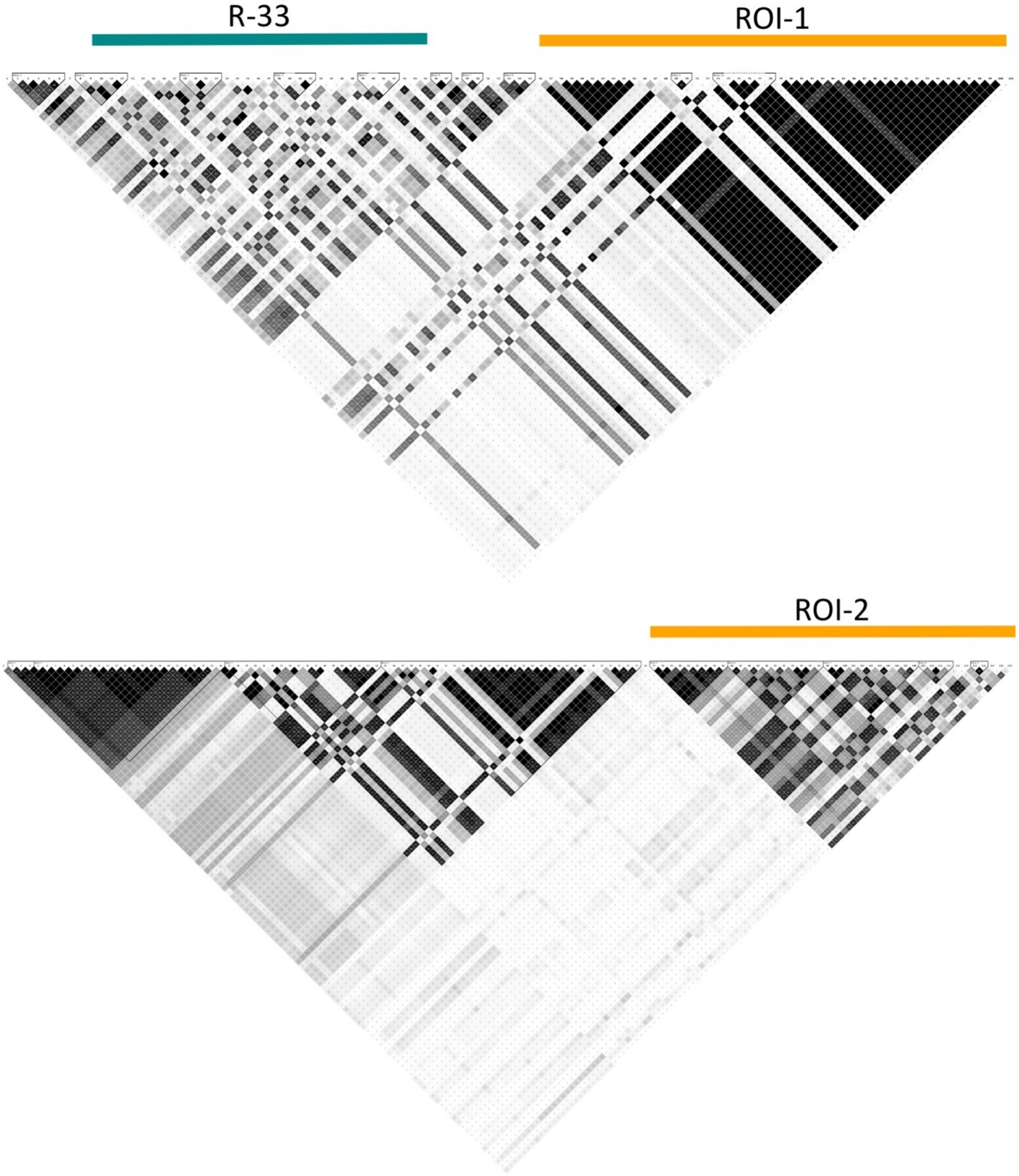

Figure S14. Linkage disequilibrium (LD) in regions of interest (ROIs). ROIs are indicated by orange bars. A) LD in scaffold 33 which contains both R-33 (denoted by a teal bar) and ROI-1. B) LD in scaffold 26, which contains ROI-2.

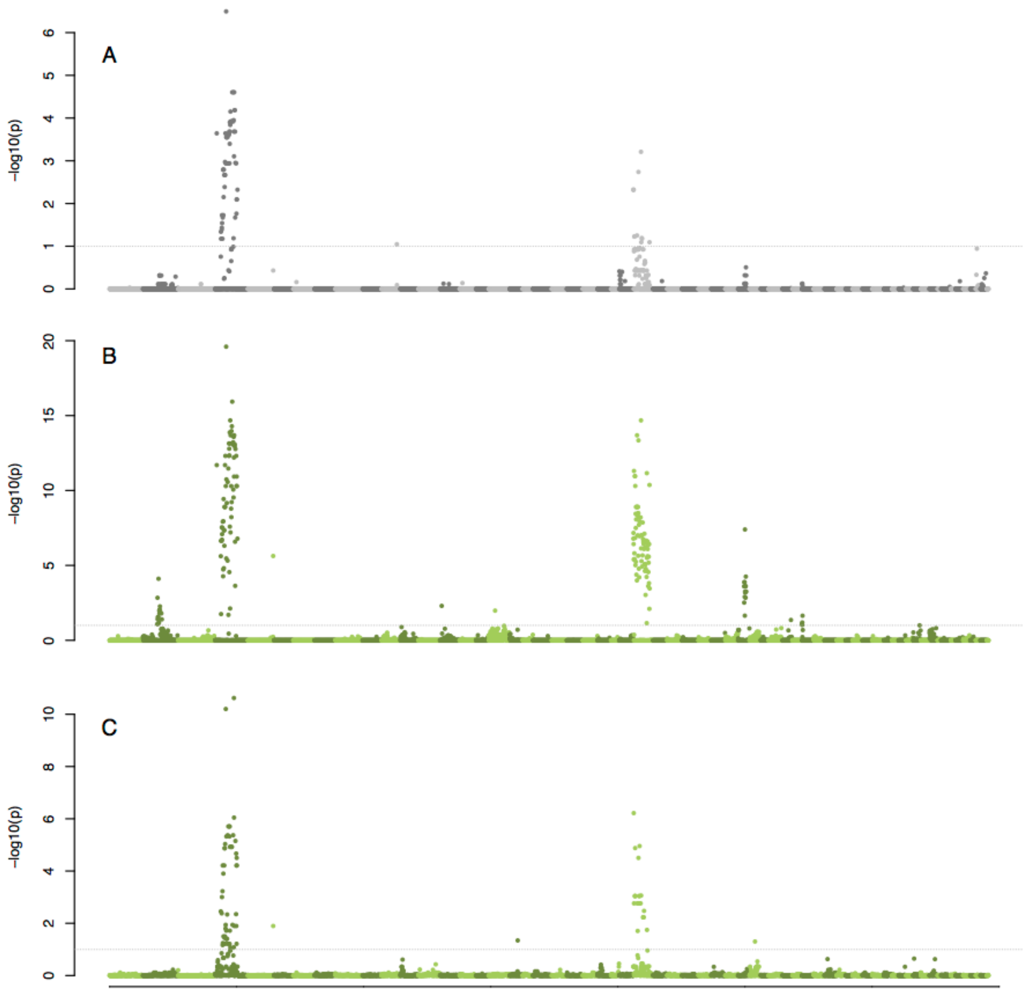

Figure S15. Allele frequency differences between isolates of opposite mating types in the *in vitro* F_1, field F_1, and field inbred subpopulations. Negative $\log_{10}$-transformed, false-discovery rate (FDR) corrected *P*-values ordered by scaffold and physical position, from the Fisher's exact test of allele frequency differences between A1 and A2 isolates in the (A) *in vitro* F_1, (B) field F_1, and (C) field inbred subpopulations. SNPs above the gray lines in A-C were significant at a 10% FDR threshold.

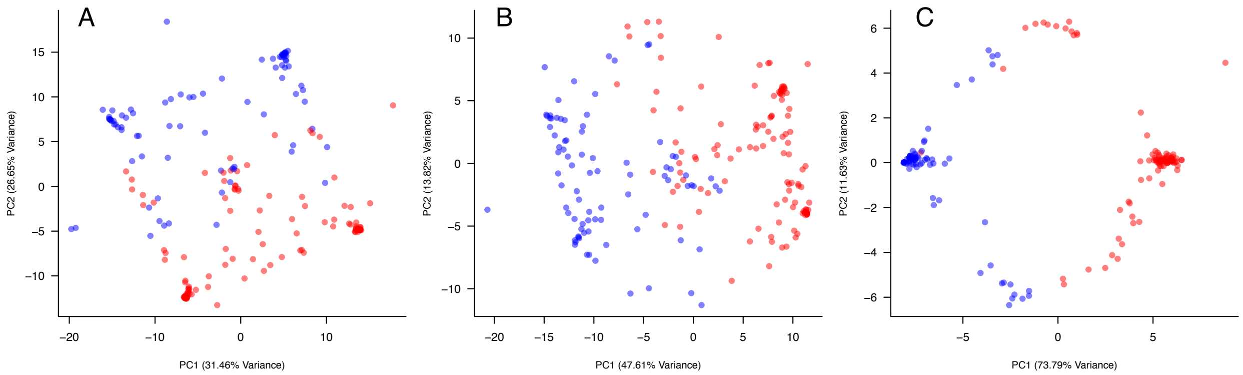

Figure S16. Principal component analysis (PCA) in the mating type region (MTR). PCA of all *in vitro* and field isolates using the: A) 293 SNPs in the MTR; B) 184 significantly differentiated SNPs in the field F_1; and the C) 51 SNPs significantly differentiated in both the field F_1 and inbred subpopulations.

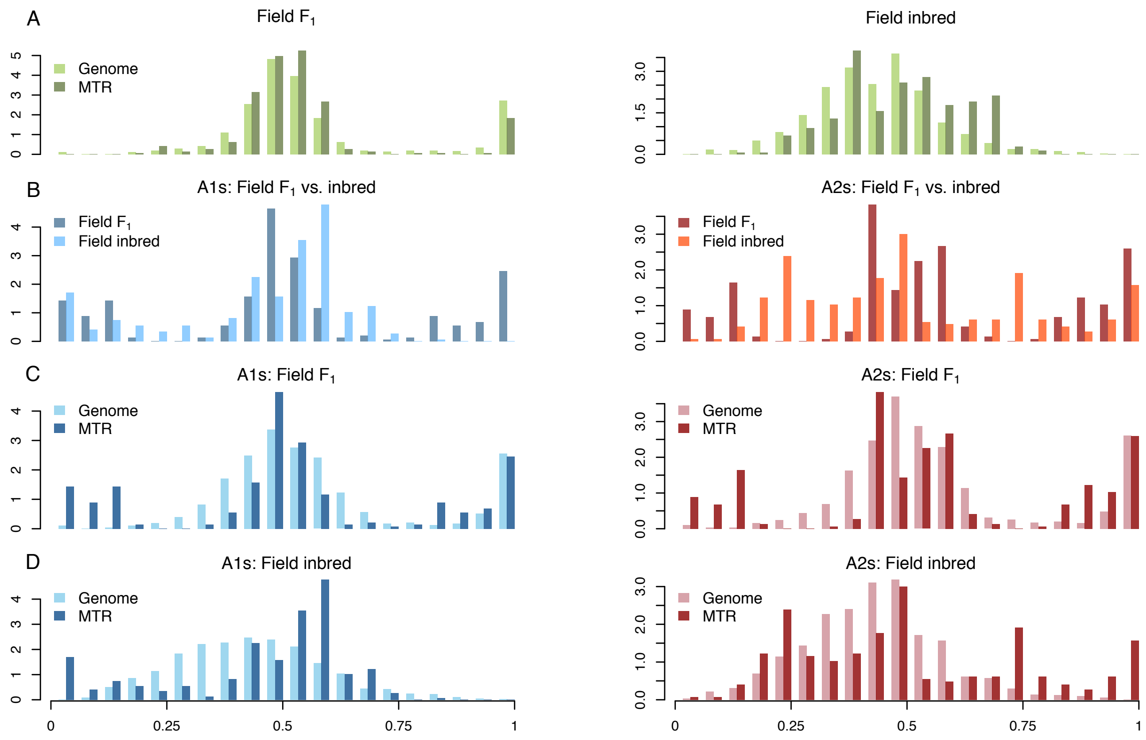

Figure S17. Heterozygosity in the mating type region (MTR) compared to the rest of the genome. Histograms of SNP heterozygosity in the MTR relative to the genome, represented by density distributions, for the: A) field F_1 and field inbred isolates; B) A1 and A2 field F_1 vs. field inbred isolates in the MTR; C) A1 and A2 field F_1 isolates in the MTR relative to the genome; and D) A1 and A2 field inbred isolates in the MTR relative to the genome.

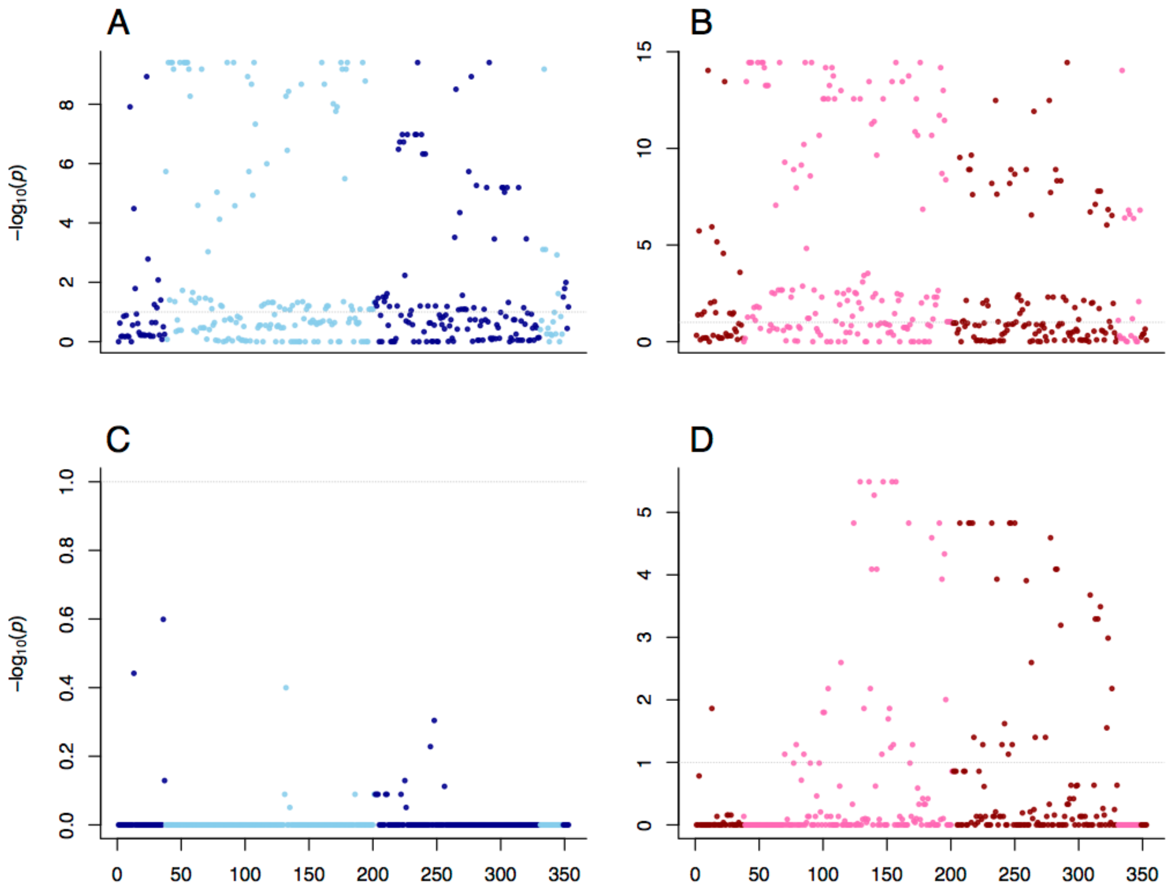

Figure S18. Heterozygote excess in the mating type associated sub-regions. Exact test of heterozygote excess in all five mating type associated sub-regions (*n*=353 SNPs), ordered by scaffold (2, 4, 27, 34, and 40) and position within scaffold. For: A) A1 field F_1 isolates; B) A2 field F_1 isolates; C) A1 field inbred isolates; and D) A2 field inbred isolates.
