# Supplementary material for: Temporal Genetic Dynamics of an Experimental, Biparental Field Population of Phytophthora capsici
Source: Front Genet. 2017 Mar 13;8:26. doi: 10.3389/fgene.2017.00026 (PMC5347166; doi:10.3389/fgene.2017.00026)
Supplement: Supplementary file 5 [file Data_Sheet_5.PDF]

	Sample					
Variables	2009	2010	2011	2012	2013	Total field
	Not clone-corrected					
MT						
A1	14	21	21	11	17*	84
A2	26	34	32	24	31	147
$\chi 2$ *P*-value**	**0.058**	**0.080**	0.131	**0.028**	**<0.001**	**<0.001**
Total	40	55	53	35	48	231
	Clone-corrected					
MT						
A1	13	16	18	9	9	65
A2	23	23	27	7	14	94
$\chi 2$ *P*-value**	**0.096**	0.262	0.180	0.617	0.297	**0.021**
Total	36	39	45	16	23	159
Unique genotypes (%)	90.00	70.91	84.91	45.71	47.92	68.83

*Excluding the isolate, 13PF_29A, which exhibited a skewed allele depth ratio distribution.

**Bold indicates significance (α<0.1)

Table S1. Counts of A1 and A2 mating types among non-clone-corrected and clone-corrected isolates with respect to year.

Replicate	Short ID	Culture date	Mycelial culture date	DNA extraction date	Sequence date	Sequence Plate	Mean IBS	Culture group	Rep Group
				**A1 parent**					
1	Pcap4777	pre-7/2013	pre-7/2013	pre-7/2013	7/31/13	C270BACXX_3	97.65%	-	-
2	664_1	pre-9/2013	pre-9/2013	9/30/13	5/27/14	C4B1JACXX_8	98.22%	-	-
3	0664_1_T25	2/17/14	pre-9/2014	pre-9/2014	9/19/14	C507CACXX_7	98.01%	-	-
4	0664_1_T26	4/7/14	pre-9/2014	pre-9/2014	9/19/14	C507CACXX_7	97.72%	-	-
5	664_T29_1a	12/8/14	3/16/15	3/23/15	6/12/15	C6H57ANXX_7	98.48%	e	e
6	664_T29_1b	12/8/14	3/16/15	3/23/15	6/12/15	C6H57ANXX_7	98.44%	e	e
7	664_T29_2a	12/8/14	3/16/15	3/23/15	6/12/15	C6H57ANXX_7	98.49%	e	f
8	664_T29_2b	12/8/14	3/16/15	3/23/15	6/12/15	C6H57ANXX_7	98.44%	e	f
9	0664_T29_1	12/8/14	3/16/15	3/23/15	8/27/15	C6P86ANXX_1	98.47%	e	-
10	0664_T29_2	12/8/14	3/16/15	3/23/15	8/27/15	C6P86ANXX_1	98.44%	e	-
11	664_T29_1a	12/8/14	3/16/15	3/23/15	8/3/15	C6RD8ANXX_1	98.35%	e	e
12	664_T29_1b	12/8/14	3/16/15	3/23/15	8/3/15	C6RD8ANXX_1	98.25%	e	e
13	664_T29_2a	12/8/14	3/16/15	3/23/15	8/3/15	C6RD8ANXX_1	98.18%	e	f
14	664_T29_2b	12/8/14	3/16/15	3/23/15	8/3/15	C6RD8ANXX_1	98.19%	e	f
				**A2 parent**					
1	Pcap4778	pre-7/2013	pre-7/2013	pre-7/2013	7/31/13	C270BACXX_3	97.92%	-	-
2	6180_4	pre-9/2013	pre-9/2013	9/30/13	5/27/14	C4B1JACXX_8	97.99%	-	-
3	06180_4_T21	pre-9/2013	pre-9/2013	9/30/13	9/19/14	C507CACXX_7	97.71%	-	-
4	6180_0217a	2/17/15	3/16/15	3/23/15	6/12/15	C6H57ANXX_7	98.30%	c	d
5	6180_0217b	2/17/15	3/16/15	3/23/15	6/12/15	C6H57ANXX_7	98.39%	c	d
6	6180_012815a	1/28/15	3/16/15	3/23/15	6/12/15	C6H57ANXX_7	98.39%	d	e
-	6180_012815b	1/28/15	3/16/15	3/23/15	6/12/15	C6H57ANXX_7	-	-	-
7	06180_128	1/28/15	3/16/15	3/23/15	8/27/15	C6P86ANXX_1	98.09%	d	-
8	6180_0217a	2/17/15	3/16/15	3/23/15	8/3/15	C6RD8ANXX_1	98.03%	c	d
9	6180_0217b	2/17/15	3/16/15	3/23/15	8/3/15	C6RD8ANXX_1	98.24%	c	d
10	6180_012815a	1/28/15	3/16/15	3/23/15	8/3/15	C6RD8ANXX_1	98.24%	d	e
11	6180_012815b	1/28/15	3/16/15	3/23/15	8/3/15	C6RD8ANXX_1	98.12%	d	e

Table S2. Replicates of the parental isolates.

Region	Scaffold	Minimum Significant Position (bp)	Maximum Significant Position (bp)	ROI size (bp)	Significant SNPs (#)	Significant SNPs in region (%)	Density of Significant SNPs (SNP/kb)	Linkage group
R-8	8	341,231	1,121,521	780,290	6	5.22	0.01	8
R-19	19	704,365	888,453	184,088	23	67.65	0.12	16
R-26	26	101,162	618,561	517,399	14	16.28	0.03	8
R-33	33	54,561	181,690	127,129	17	45.95	0.13	16
R-35	35	142,871	508,327	365,456	22	31.88	0.06	16
R-55	55	85,182	383,472	298,290	29	59.18	0.1	16

Table S3. Regions of differentiation between field F_1 and *in vitro* F_1 isolates associated with incidence of Mendelian errors.

	Genotype counts*											Haplotype counts***			
	H1/H2	H3/H4	H1/H3	H1/H4	H2/H3	H2/H4	*H1/H1*	*H2/H2*	*H3/H3*	*H4/H4*	*Unknown***	**H1**	**H2**	**H3**	**H4**
**R-8 (sc8)**															
Field F1			27	25	18	22	*1*	*1*	*1*		9	54	42	47	47
**Observed Parents**	**A1**								***A2***			1	1	1	0
in vitro F1			12	6	15	4	*1*		*3*			20	19	33	10
**R-26 (sc26)**															
Field F1			15	24	27	32		*2*	*1*		3	39	63	44	56
**Observed Parents**	**A1**								***A2***			1	1	1	0
in vitro F1			13	5	14	5	*1*		*3*			20	19	33	10
**R-33 (sc33)**															
Field F1	*1*		27	28	20	18	*1*	*1*	*2*		6	58	41	51	46
**Observed Parents**		**A2**					***A1***					1	1	1	0
in vitro F1			13	23	0	1	*3*			*1*		42	1	13	26
**R-35 (sc35)**															
Field F1	*1*		17	33	21	24	*1*	*1*	*2*	*1*	3	53	48	42	59
**Observed Parents**							***A1***		***A2***			1	1	1	0
in vitro F1			19	12	0	0	*5*		*2*		3	41	0	23	12
**R-55 (sc55)**															
Field F1	*1*		21	30	18	25	*2*		*3*	*1*	3	56	44	45	57
**Observed Parents**		**A2**					***A1***					1	1	1	0
in vitro F1			23	11	0	0	*5*		*2*			44	0	27	11

*Outlined cells indicate the expected genotypes for the Field F1, in vitro F1, and parental isolates. Italic font and no outline indicate where observed data did not cohere with expectations.

**Where the haplotype designation was unclear, i.e. due to recombination, the isolate was classified as Unknown. These isolates are indicated in S10 and S12 Figs for R-26 and R-35. Phasing also shown for R-33, but denoted haplotypes are for ROI-1, not R-33.

***Haplotype counts do not necessarily sum to the sample size due to the Unknown isolates.

Table S4. Genotype and haplotype counts for regions of differentiation between the field F_1 and *in vitro* F_1.

Region	Scaffold	Minimum Significant Position (bp)	Maximum Significant Position (bp)	ROI size (bp)	Significant SNPs (#)	Significant SNPs in region (%)	Density of Significant SNPs (SNP/kb)	Linkage group
-	7	422,903	1,260,693	837,790	9	7.09	0.01	1 & 13
-	18	720,911	895,992	175,081	3	18.75	0.02	2
-	37	183,312	511,376	328,064	5	6.58	0.02	3
-	6	58,320	945,375	887,055	20	20.2	0.02	4
-	10	375,319	1,112,682	737,363	7	5.83	0.01	5
ROI-2	21	615,928	906,387	290,459	29	67.44	0.1	5
-	24	401,564	662,972	261,408	9	26.47	0.03	5
-	52	6,287	133,781	127,494	5	20	0.04	5
-	63	66,589	303,371	236,782	7	21.21	0.03	5
-	68	49,163	293,159	243,996	13	28.89	0.05	5
-	8	1,204	1,107,380	1,106,176	19	12.67	0.02	8
-	22	515,495	704,555	189,060	8	23.53	0.04	8
-	58	107,853	313,193	205,340	8	16.33	0.04	8
-	62	181,179	212,989	31,810	3	25	0.09	8
-	2	295,579	1,796,377	1,500,798	15	6.55	0.01	10 & 13
-	3	45,932	836,688	790,756	5	3.76	0.01	10 & 13
-	34	320,234	405,897	85,663	9	42.86	0.11	10
-	53	22,057	411,035	388,978	9	15.79	0.02	12
-	36	112,937	484,604	371,667	13	37.14	0.03	13
-	20	143,241	806,490	663,249	20	15.5	0.03	16
ROI-1	33	47,384	560,094	512,710	52	55.32	0.1	16
-	55	23,126	372,172	349,046	9	16.07	0.03	16

Table S5. Regions of differentiation between the field F_1 and inbred subpopulations.

	Genotype counts*							Haplotype counts			Haplotype (%)***		
	H1/H1	H3/H4	H1/H3	H1/H4	H3/H3	H4/H4	*Unknown***	**H1**	**H3**	**H4**	**H1**	**H3**	**H4**
**Observed Parents**	**A1**	**A2**						2.0	1.0	1.0	0.5	0.25	0.25
**Field F1**													
2009	2		15	15	*1*		3	34	17	15	47.22	23.61	20.83
2010			20	19				39	20	19	50.00	25.64	24.36
2011	1		15	12	*1*			29	17	12	50.00	29.31	20.69
**Total**	**3**		**50**	**46**	**2**		**3**	102	54	46	49.04	25.96	22.12
**Field inbred**													
2011	4		7	3				18	7	3	64.29	25.00	10.71
2012	6		6		4			18	14	0	56.25	43.75	0.00
2013	5	1	12	1	4			23	21	2	50.00	45.65	4.35
**Total**	**15**	**1**	**25**	**4**	**8**			59	42	5	55.66	39.62	4.72

*Outlined boxes denote the expected genotypes for the parents, Field F1 and Field inbred, assuming simple Mendelian inheritance.
**Where haplotype was unclear, i.e. due to recombination between distinct haplotypes, the isolate was classified as Unknown. These isolates are indicated in S11 Fig.
***Haplotype frequencies do not necessarily sum to one due to the Unknown genotypes.

Table S6. Genotype and haplotype counts in ROI-1 in the field population with respect to year and subpopulation (F_1 vs. inbred).

	Genotype counts*							Haplotype counts			Haplotype (%)***		
	H1/H1	H3/H4	H1/H3	H1/H4	H3/H3	H4/H4	*Unknown***	**H1**	**H3**	**H4**	**H1**	**H3**	**H4**
**Observed Parents**	**A1**	**A2**						2	1	1	0.5	0.25	0.25
**Field F1**													
2009			21	15				36	21	15	50.00	29.17	20.83
2010			24	15				39	24	15	50.00	30.77	19.23
2011			21	8				29	21	8	50.00	36.21	13.79
**Total**			**66**	**38**				104	66	38	50.00	31.73	18.27
**Field inbred**													
2011	9		3	1			1	22	3	1	78.57	10.71	3.57
2012	9		4	3				25	4	3	78.13	12.50	9.38
2013	10	5	1	6			1	27	6	11	58.70	13.04	23.91
**Total**	**28**	**5**	**8**	**10**			2	74	13	15	69.81	12.26	14.15

*Outlined boxes denote the expected genotypes for the parents, Field F1 and Field inbred, assuming simple Mendelian inheritance.

**Where haplotype was unclear, i.e. due to recombination between distinct haplotypes, the isolate was classified as Unknown.

***Haplotype frequencies do not necessarily sum to one due to the Unknown genotypes.

Table S7. Genotype and haplotype counts in ROI-2 in the field population with respect to year and subpopulation (F_1 vs. inbred).

Scaffold	Minimum Position (bp)	Maximum Position (bp)	Size (bp)	Number of SNPs in region	Total SNPs in scaffold
Field F1					
2	857,230	1,027,986	170,756	17	276
4	78,389	936,543	858,154	69	258
27	1,260	559,598	558,338	80	156
34	373,020	419,673	46,653	14	90
40	53,533	62,968	9,435	4	92
Field inbred					
4	204,373	896,170	691,797	53	258
27	8,102	506,357	498,255	20	156

Table S8. Regions of differentiation between isolates of opposite mating types in the field F_1 and inbred subpopulations.
